# Registration accuracy with the low dose kilovoltage cone-beam CT: A phantom study

**DOI:** 10.1259/bjro.20190028

**Published:** 2019-08-29

**Authors:** Yoshiki Takei, Hajime Monzen, Kenji Matsumoto, Kohei Hanaoka, Mikoto Tamura, Yasumasa Nishimura

**Affiliations:** 1 Department of Radiology, Kindai University Nara Hospital, Nara, Japan; 2 Graduate School of Medical Science, Department of Medical Physics, Kindai University, Osaka, Japan; 3 Department of Radiation Oncology, Faculty of Medicine, Kindai University, Osaka, Japan

## Abstract

**Objective:**

The aim of this study was to investigate low-dose kilovoltage cone-beam CT (kV-CBCT) for image-guided radiotherapy, with a particular focus on the accuracy of image registration with low-dose protocols.

**Methods:**

Imaging doses were measured with a NOMEX semiconductor detector positioned at the front of head, thorax, and pelvis human body phantoms while kV-CBCT scans were acquired at different tube currents. Aspects of image quality (spatial resolution, noise, uniformity, contrast, geometric distortion, and Hounsfield unit sensitivity) and image registration accuracy using bone and soft tissue were evaluated.

**Results:**

With preset and the lowest tube currents, the imaging doses were 0.16 and 0.08 mGy, 5.29 and 2.80 mGy, and 18.23 and 2.69 mGy for head, thorax, and pelvis, respectively. Noise was the only quality aspect directly dependent on tube current, being increased by 1.5 times with a tube current half that of the preset in head and thorax, and by 2.2 times with a tube current 1/8 of the preset in the pelvis. Accurate auto-bone matching was performed within 1 mm at the lowest tube current. The auto-soft tissue matching could not be performed with the lowest tube current; however, manual-soft tissue matching could still be performed within 2 mm or less.

**Conclusion:**

Noise was the only image quality aspect dependent on the imaging dose. Auto-bone and manual-soft tissue matching could still be performed at the lowest imaging dose.

**Advances in knowledge:**

When optimizing kV-CBCT imaging dose, the impact on bone and soft tissue image registration accuracy should be evaluated.

## Introduction

The availability of high-precision radiation therapy techniques such as intensity modulated radiotherapy (IMRT), volumetric modulated arc therapy (VMAT), and image-guided radiotherapy (IGRT) has spread rapidly, associating with improvements in the geometric accuracy of patient positioning in radiotherapy. This rapid increase in availability can lead to an increase in the imaging frequency. However, a problem with IGRT is the increased dose to normal tissue around the target, due to the increased imaging frequency; this is in addition to the treatment dose.^1,2^ Furthermore, as the imaging fields are much larger than the radiotherapy field, the imaging dose presents an unnecessary dose to tissue outside the radiotherapy field. In high-dose imaging procedures such as cone-beam CT (CBCT), the daily imaging dose to tissue outside the treated volume may be comparable to the stray dose in these tissues from the radiotherapy procedures.^3^ Epidemiological studies have shown an increased skin cancer risk outside the radiotherapy field, even at relatively low skin doses.^4^ In pediatric patients, the use of standard CBCT imaging protocols often results in an unnecessarily excessive dose, because of the smaller patient body size.^5^ Therefore, in line with the ALARA (“as low as reasonably achievable”) principle, the adequate management of the imaging dose is necessary for IGRT.^6^

The dose management program of the American Association of Physicists in Medicine Task Group 75 advocates evaluation, reduction, and optimization of the imaging dose.^7^ The IGRT imaging dose has been evaluated in various ways, including with an ion chamber, thermoluminescent dosimeter (TLD), and radiochromic film.^8–11^ The methods available for reducing the imaging dose include reduction of the mAs value, the projection number used for reconstruction, and the scan length, the use of a filter, and changing of the imaging angle.^12–17^ The mAs value has a linear correlation with imaging dose,^1^ and it can be adjusted at the time of imaging, with the imaging dose being estimated simultaneously. However, a reduction in the imaging dose increases noise, which leads to deterioration in image quality.^8^ Therefore, it is necessary to evaluate the influence of this deterioration in image quality on the image registration accuracy, to ensure optimization of the imaging dose. Nevertheless there have been several studies on optimization of imaging dose,^18,19^ however, there is no study that comprehensively evaluated image registration accuracy including manual-soft tissue matching.

The purpose of this study was therefore to investigate low-dose kilovoltage CBCT (kV-CBCT), and to determine whether it could be used to perform accurate image registration in IGRT. Several image quality aspects and the resultant registration accuracy of bone and soft tissue techniques were evaluated using various kV-CBCT X-ray currents.

## Methods and materials

### Measurement of imaging dose

**Figure 1.**
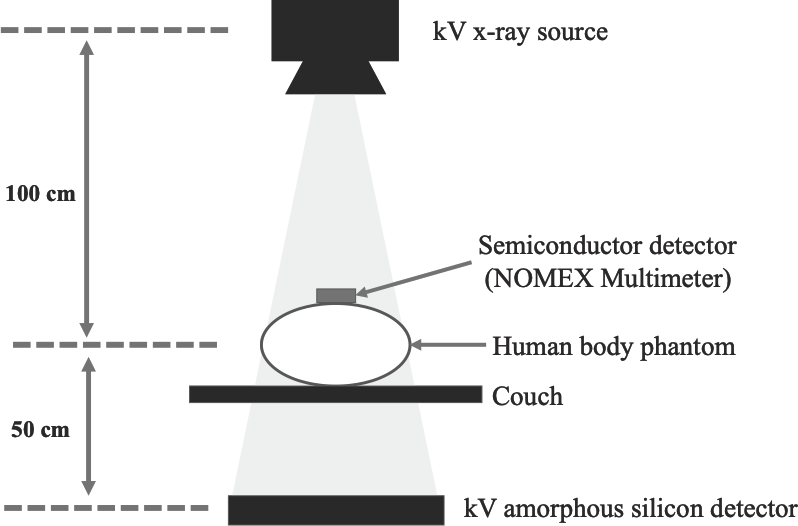
A schematic diagram of the measurement geometry for imaging dose with kilovoltage cone-beam CT.

The kV-CBCT imaging doses were measured using a commercially available NOMEX semiconductor detector (NOMEX Multimeter, PTW, Freiburg, Germany) positioned at the front of human body phantoms of the head, thorax, and pelvis (Kyotokagaku, Kyoto, Japan) (Figure 1). The dose calibration of NOMEX has been carried out within 3 years of measurement according to the energy defined by IEC 61267, and the measurement uncertainty in the NOMEX is ±2.5% at the 95% confidence level. NOMEX is a non-connected and compact multiparameter measuring device, which can be used simply for evaluating the effect of imaging dose reduction. The kV-CBCT was performed using an On-Board Imager (OBI ver.1.5) kilovolt imaging system on a Clinac iX linear accelerator (Varian Medical Systems Inc., Palo Alto, CA). Four preset protocols (Low Dose Head, Standard Dose Head, Low Dose Thorax, Pelvis) were used in this study (Table 1).^20^ The imaging dose was reduced by decreasing the X-ray tube current from the preset value (head and thorax: 20 mA, pelvis: 80 mA), while maintaining the same X-ray millisecond. With consideration of the direction dependency of the NOMEX semiconductor detector, we measured the imaging dose with NOMEX facing upward and downward, and used the value obtained by summing up each measured value.

**Table 1.** The preset cone-beam CT (CBCT) scan protocols.

	**Low dose head**	**Standard dose head**	**Low dose thorax**	**Pelvis**
X-ray voltage (kVp)	100	100	110	125
X-ray current (mA)	10	20	20	80
X-ray millisecond (ms)	20	20	20	13
Gantry rotation (deg)	200	200	360	360
Number of projections	360	360	655	655
Exposure (mAs)	72	145	262	680
Fan type	Full fan	Full fan	Half fan	Half fan
Bow-tie filter	Full	Full	Half	Half
Default pixel matrix	384 × 384	384 × 384	384 × 384	384 × 384
Dose CTDIw (mGy)	2	3.9	4.7	17.7
Recon. filter	Standard	Sharp	Standard	Standard
Slice thickness (mm)	2.5	2.5	2.5	2.5

### Evaluation of image quality

Image quality aspects of the kV-CBCT images, including (i) spatial resolution, (ii) noise, (iii) uniformity, (iv) contrast, (v) geometric distortion, and (vi) Hounsfield Unit (HU) sensitivity, were evaluated with a Catphan 504 phantom (The Phantom Laboratory, NY) and SNC machine analysis software (SunNuclear, Melbourne, FL). The center of the Catphan 504 phantom was positioned at the isocenter. The evaluation methods for each aspect of image quality were as follows.

**Figure 2.**
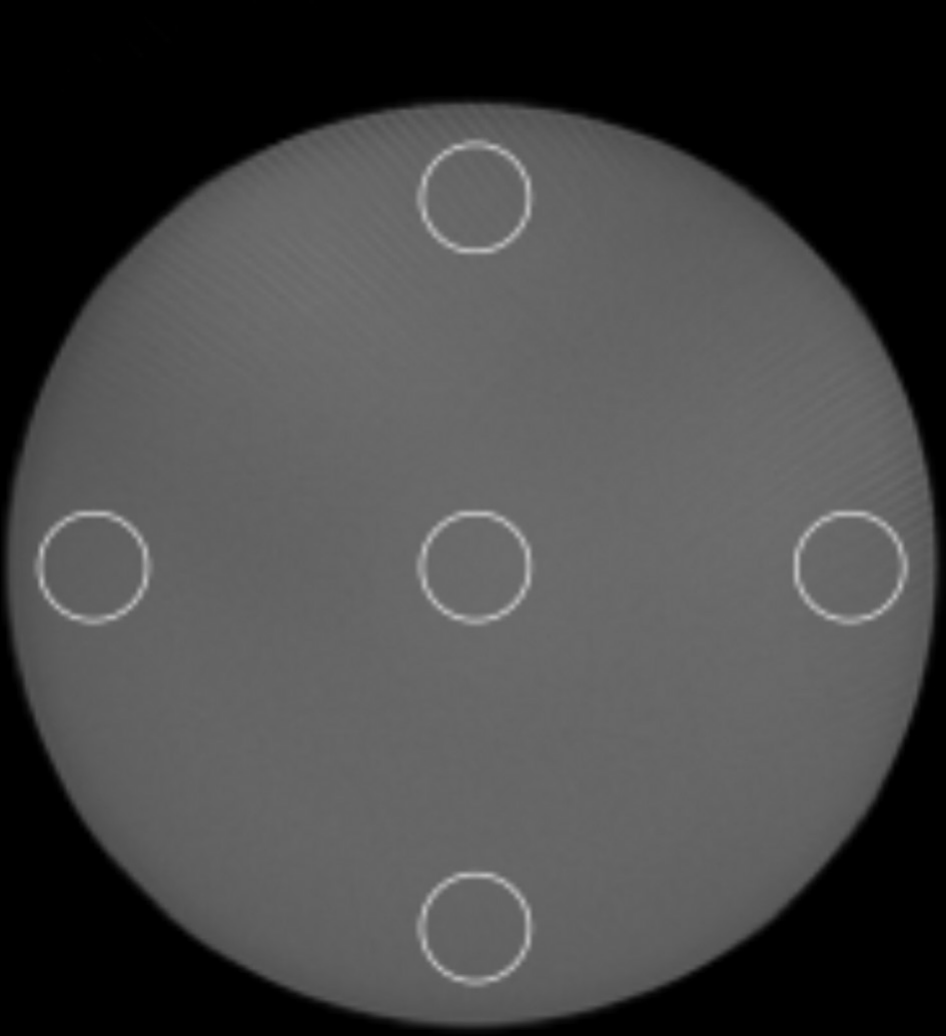
Catphan uniformity module (CTP486). ROIs were generated at the center and each of the four peripheral locations to evaluate uniformity.

- A Catphan CTP528 module with 1 to 21 line pairs per cm (lp/cm) was used to evaluate the spatial resolution according to the identifiable line pairs per cm.
- Noise was measured using a CTP486 module containing uniform material with a CT number of 20 HU. Noise was defined as the standard deviation of the background and an air region of interest (ROI) was used as the background.
- Uniformity was calculated as the difference between the maximum and minimum mean HU values of the five ROIs at the center of the phantom and at 3, 6, 9, and 12 o'clock positions around the periphery of the CTP486 module (Figure 2).^21^
- A CTP404 module with seven inserts including air (−1000 HU), polymethylpentene (PMP; −200 HU), low density polyethylene (LDPE; −100 HU), polystyrene (−35 HU), water (0 HU), acrylic (120 HU), Delrin (340 HU), and Teflon (990 HU) was used to evaluate contrast. Contrast was defined as (A−B)/A, where A is the mean gray-scale value of the Teflon ROI, and B is the mean gray-scale value of the disc background measured from an ROI between the Teflon and Delrin ROIs.
- Geometric distortion was evaluated as the maximum deviation from 50 mm for the measured distance of four holes drilled at 50 mm intervals in the CTP404 module.
- HU sensitivity was evaluated from the HU of each insert of the CTP404 module.

### Evaluation of image registration

Planning CT (pCT) images of the human body phantoms (head, thorax, and pelvis) were obtained on a CT scanner (Alexion TOSHIBA, Tokyo, Japan). The parameters included an X-ray voltage of 120 kVp, X-ray current of 200 mA, and helical pitch of 11.0. A digitally reconstructed radiograph was obtained from the radiation treatment planning system (Eclipse ver.11, Varian Medical Systems Inc., Palo Alto, CA) to act as the reference image. A rigid auto-registration was performed between the pCT image and kV-CBCT image using Offline Review image registration software (Varian Medical Systems Inc., Palo Alto, CA), to verify the accuracy of the image registration. The isocenter of the reference image was moved 1 cm along each of the three axes on the software. This auto-registration was performed three times for each exposure condition. The registrations between the preset protocol CBCT images (as shown in Table 1) and the pCT images were set to the reference accuracy, and the image registrations were evaluated according to differences in the translations along the three axes (AP, SI, LR) in comparison with the reference accuracy. The pelvic phantom contained organ-model inserts with similar HU values to the human body (prostate: 50 HU, seminal vesicle: 25 HU, bladder surface: 30 HU, bladder filling: 10 HU, rectal surface: 70 HU, rectal cavity: −800 HU), making soft tissue matching possible. The experienced radiation therapists (RTs) and medical physicists (MPs) used soft tissue matching to perform manual-registrations for the pelvic phantom, repeating these registrations three times for each exposure condition. We used the whole pelvis as the region of interest in bone matching, while in soft tissue matching, we used the soft tissue region in the pelvic cavity to exclude the effects of bone structure.

## Results

### Imaging dose

**Figure 3.**
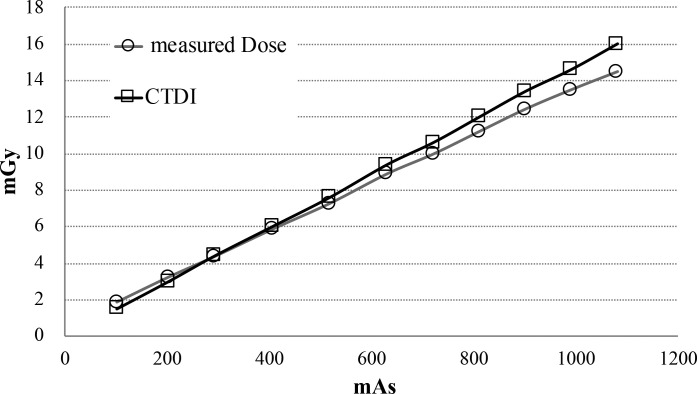
The relationship between and measurement dose by NOMEX and CTDI provided by manufacturer. The standard deviation (1SD) of the measurement by NOMEX was less than 1.0%.

The imaging doses under each condition are shown in Table 2. The imaging doses for the preset protocols of Low Dose Head, Standard Dose Head, Low Dose Thorax, and Pelvis were 0.08 ± 0.00 mGy, 0.16 ± 0.00 mGy, 5.29 ± 0.04 mGy, and 18.23 ± 0.09 mGy respectively. The imaging doses for Standard Dose Head, Low Dose Thorax, and Pelvis under minimum condition (10 mA) were 0.08 ± 0.00 mGy, 2.80 ± 0.01 mGy, and 2.69 ± 0.01 mGy respectively. A linear relationship between the mAs values and imaging doses measured with the NOMEX was observed under each exposure condition (coefficient of determination: R^2^ = 0.999). And also a linear relationship between the CTDI values provided by manufacturer and imaging doses measured with the NOMEX was observed (R^2^ = 0.999) (Figure 3). The standard deviation (1SD) of the measurement by NOMEX was <1.0%. The NOMEX was therefore considered sufficient for a simple evaluation of the effect of imaging dose reduction.

**Table 2.** Summary of the absorbed doses (mean ± standard deviation) from cone-beam CT (CBCT) measured by NOMEX at the front of the human body phantom.

**Standard dose head**			**Low dose thorax**			**Pelvis**		
mA	mAs	**Dose (mGy)**	mA	mAs	**Dose (mGy)**	mA	mAs	**Dose (mGy)**
10	73	0.08 ± 0.00	10	131	2.80 ± 0.01	10	85	2.69 ± 0.01
12.5	91	0.10 ± 0.00	12.5	164	3.37 ± 0.02	12.5	106	3.22 ± 0.01
16	116	0.13 ± 0.00	16	210	4.31 ± 0.02	16	136	3.99 ± 0.01
20	145	0.16 ± 0.00	20	262	5.29 ± 0.04	20	170	4.89 ± 0.02
						25	213	6.01 ± 0.03
Low dose head						32	272	7.60 ± 0.03
mA	mAs	Dose (mGy)				40	340	9.34 ± 0.06
10	72	0.08 ± 0.00				50	425	11.61 ± 0.05
						63	536	14.61 ± 0.05
						80	680	18.23 ± 0.09

### Image quality

**Figure 4.**
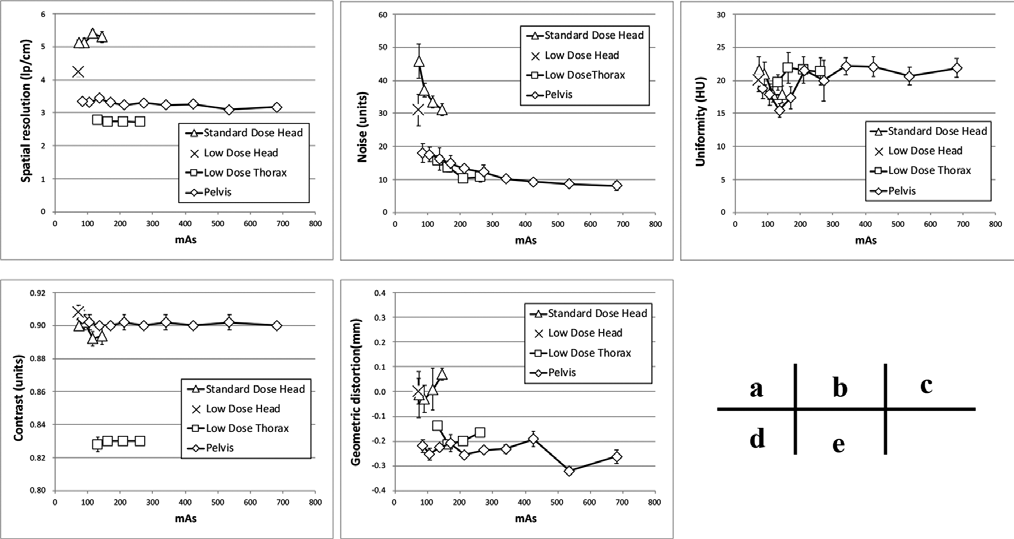
Results of the image quality test; (a) spatial resolution, (b) noise, (c) uniformity, (d) contrast, (e) geometric distortion.

The results of the image quality assessment are shown in Figure 4. Spatial resolution was the highest in the Standard Dose Head protocol, followed by Low Dose Head, Pelvis, and Low Dose Thorax (Figure 4a), although the spatial resolutions of the Standard Dose Head, Low Dose Head, and Pelvis were lower than the accepted values.

Noise was the highest with the Standard Dose Head under minimum condition (Figure 4b), while it was the same for the Standard Dose Head and the Low Dose Head preset protocols. Uniformity was similar across all protocols (Figure 4c). The Low Dose Thorax showed the lowest contrast, while the other protocols showed similar levels of contrast (Figure 4d). Geometric distortions were the same for all protocols (Figure 4e). Among the image quality items, only the noise depended on the mAs value, increasing as the mAs decreased. With the Standard Dose Head and Low Dose Thorax settings, the noise increased by 1.5 times with a dose half that of the preset protocol. With the Pelvis setting, the noise increased by 1.3 times with a dose half that of the preset protocol, and 2.2 times with a 1/8 dose. The HU sensitivity did not depend on the mAs value, and was within ±15 HU of the preset value for all inserts.

### Image registration

**Figure 5.**
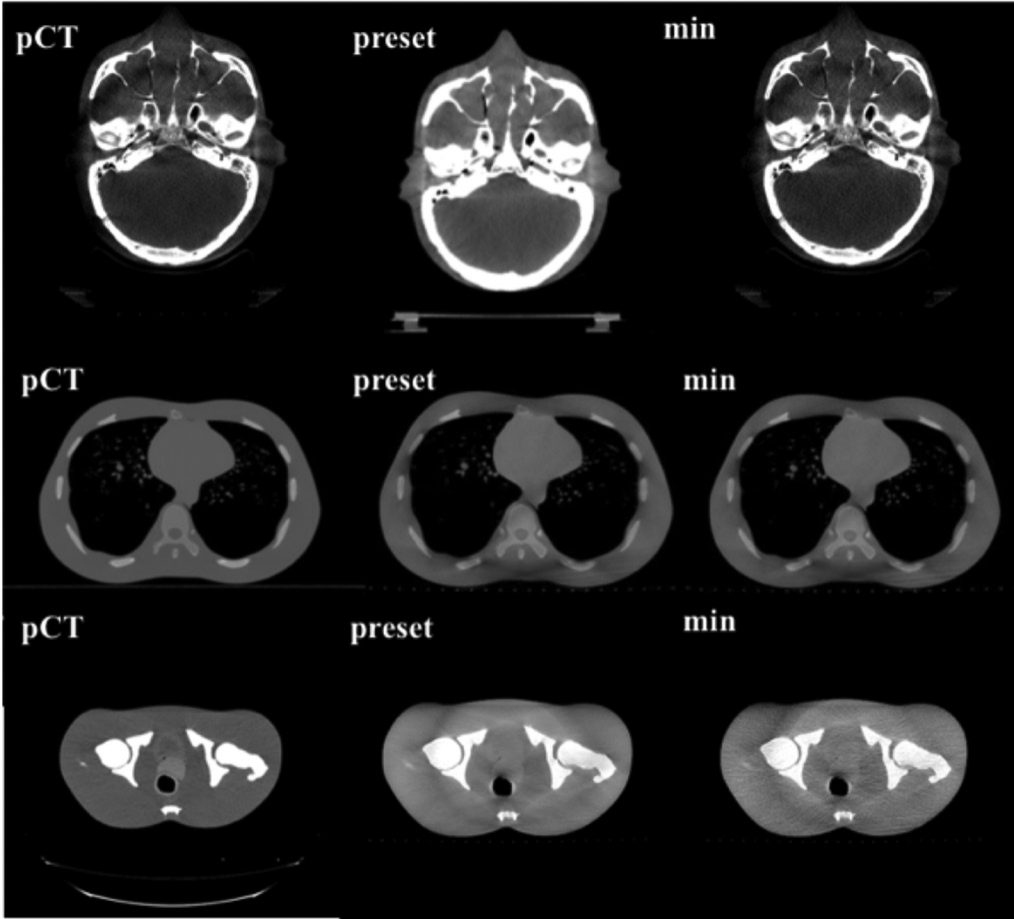
Phantom images (head, thorax, pelvis) from the planning CT (pCT), CBCT preset protocol (preset), and minimum dose condition (min).

Images of each phantom under pCT, the CBCT preset protocols, and CBCT minimum dose conditions are shown in Figure 5. For each phantom, the noise component increased with the low imaging dose, although the bone structure could still be visually recognized. Auto-bone matching for the head, thorax, and pelvis could be performed within 1 mm under all conditions, even with the minimum mAs setting.

**Figure 6.**
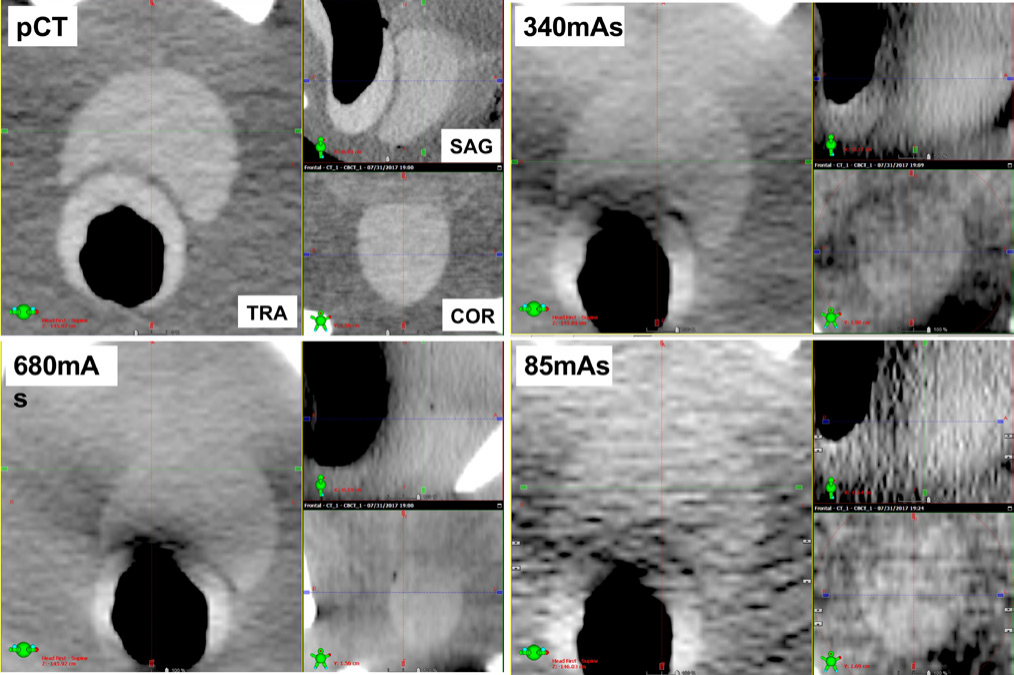
Three-section images (transvers, sagittal, coronal) inside the pelvic cavity of the pelvic phantom from the planning CT (pCT), CBCT preset condition (680 mAs), half preset condition (340 mAs), and minimum dose condition (85 mAs).

**Figure 7.**
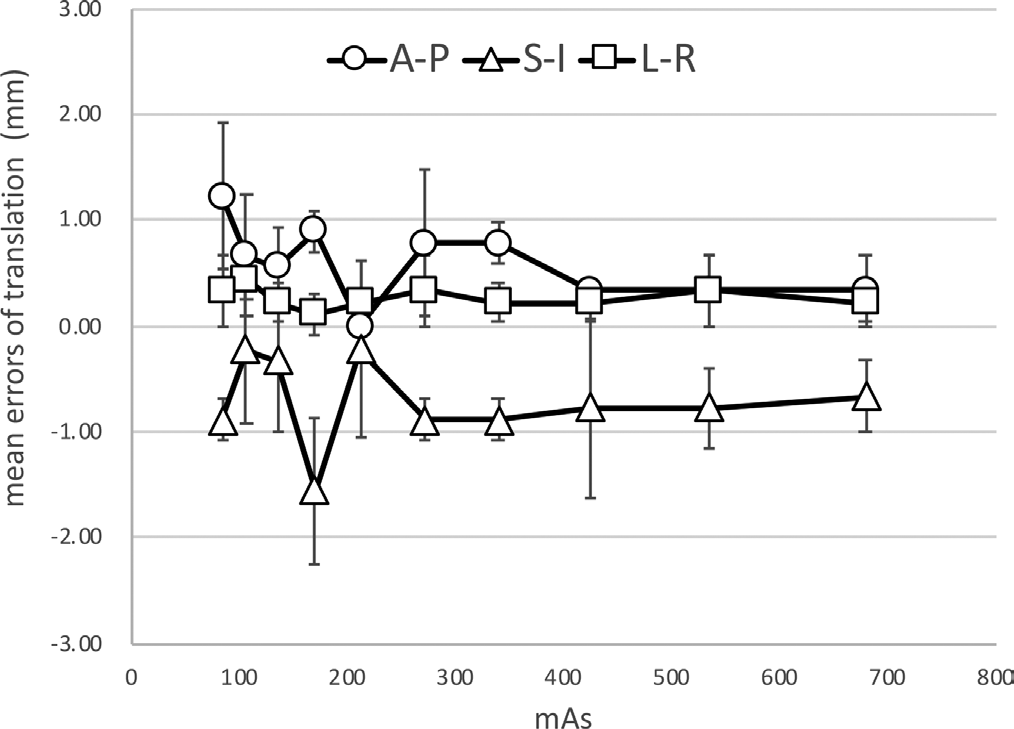
Results of the soft tissue matching by manual-registration for the pelvic phantom. Error bars represent one standard deviation.

Example images (transvers, sagittal, and coronal images) of the inside of the pelvic cavity of the pelvic phantom acquired at various reduced doses are shown in Figure 6. In the soft tissue matching, the boundary between the prostate and bladder was unclear because of the image noise component, and this boundary was difficult to identify in the sagittal section. Furthermore, the auto-matching function could not be performed accurately as the soft tissue contrast was unclear. However, even at an imaging dose of 10 mA, the boundary between the prostate and rectum could be visually recognized, and the manual- soft tissue matching was performed by the experienced RTs and MPs within 2 mm (Figure 7).

## Discussion

In this study, we optimized the imaging dose while maintaining a high level accuracy in the image registration. We demonstrated that accurate image registration can be performed within 1 mm using auto-bone matching, even if the imaging dose was reduced to half that of the clinical imaging protocol for the head and thorax, and to 1/8 of the clinical imaging protocol for the pelvis. In addition, the experienced RTs or MPs could perform accurate soft tissue matching within 2 mm or less, as shown in Figure 7.

Several studies have evaluated reductions to the imaging dose of IGRT; however, only a few reports have reported on definite reductions to the imaging dose and their influence on image quality and image registration accuracy. Lu et al reduced the imaging dose to 1/6 of the preset value by reducing the number of projections and evaluated the quantitative image quality according to three aspects: spatial resolution, low-contrast resolution, and uniformity, and their influence on the automated image registration accuracy.^13^ They concluded that low-contrast visibility was worse when the projection numbers were reduced, with this being due to increased noise and streak artifacts, and they therefore recommended reducing noise and enhancing soft tissue contrast for soft tissue matching. However, a quantitative evaluation of image noise was not performed. We investigated image quality in more detail, and demonstrated that only the image noise depended on the imaging dose, with the other factors being independent of the scan protocols, as shown in Figure 4. The spatial resolution was stable, even though the imaging dose was reduced in all protocols, and we consider that the ability to discriminate between different tissues was unaffected in the high contrast region, as shown in Figure 4a.

It is expected that image registration can be carried out using the lowest imaging dose as it is the setting provided and allowed by the manufacturer. However, when optimizing the imaging dose in IGRT, it is necessary to evaluate the influence of the deterioration of image quality on the image registration accuracy. In the study of Langmack et al, radiographers performed a visual image-quality assessment of clinical images of the prostate, and reduced the imaging dose to 80% by reducing the mAs. However, they did not consider the effect of dose reduction on the registration results, only visually evaluating the image quality at the image registration.^12^ By evaluating the results of the image registration, we identified the minimum imaging dose that still allowed image registration accuracy for IGRT to be maintained. Sykes et al evaluated the impact of reducing the projection numbers on image quality and image registration, but image registration was performed only on the head, which did not require soft tissue matching.^14^ Reduction of the imaging dose can lead to deterioration of the visibility of soft tissue in particular, and may affect soft tissue matching accuracy. In cases where accurate auto-registration cannot be performed, or when a non-rigid image registration is required, manual-registration with visual inspection is necessary. Barder et al evaluated image registration accuracy for bone auto-matching at different mAs.^18^ However, auto- or manual-matching for the soft tissues is not performed. Kuo et al evaluate the quality, dose, and registration of CBCT images acquired with different angular range and angular separation.^19^ They performed auto-matching using a simple shape target in the evaluation of alignment accuracy. In this study, we evaluated both auto-bone matching and manual-soft tissue matching by experienced RTs and MPs. For the bone matching, we confirmed that the bone structure could be visually recognized even though the imaging dose was reduced, ensuring that automated registration can be successfully performed, even with the lowest-dose imaging conditions, as shown in Figure 5. However, the automated soft tissue matching could not be performed at the lowest-dose protocols because of the image noise, as shown in Figure 6. In the Pelvis protocol, the noise increased by 1.3 times with a dose half that of the preset protocol, and 2.2 times with a 1/8 dose, as shown in Figure 4b. In the sagittal image, the soft tissue contrast was low in the SI direction of the prostate, as it was strongly affected by noise, which lead to large inter observation variations in the SI direction. However, the manual-soft tissue matching by the experienced RTs and MPs could be performed with an accuracy within 2 mm, even at the lowest dose, as shown in Figure 7. This level of accuracy can be tolerated in consideration of the PTV margin.

This study has several limitations, including imaging dose and the use of a rigid phantom, as actual patients are non-rigid and vary in physique. As a clinical practice, we have used the low-dose CBCT to the patients and been able to perform the accurate image registration. The skin dose measurement in this study is not cleared to relate to patient dose for example effective dose or CT dose (CTDI). However, we performed the stability, precision and accuracy of the NOMEX for dose measurements to obtain the relation between skin dose and CTDI. Gardner et al evaluated image quality changes on CBCT due to differences in physique. The contrast-to-noise ratio (CNR) can depend on physique, and HU and uniformity are affected by half-scan.^22^ The scan protocol needs to be carefully considered according to the physique of the patient, the scan mode, and the reference for the registration. Furthermore, there is a correlation between physique and imaging dose, and the smaller the physique, the greater the imaging dose.^5^ As the smaller physique is larger in imaging dose change than the larger one, the dose should be actively reduced for patients with a small physique, such as children, females, and young people. The physique of the patient can cause the variation of image quality and it may make impossible to evaluate the accurate registration for actual patients. Therefore, as the first step, we have shown the possibility of specific dose reduction using phantom in this study. Further study for the actual patients may be needed to evaluate the dependence of the physique to the registration accuracy and image quality with low-dose imaging dose as the next step. In this study, we used the only one CBCT system. Further study is needed for the multi CBCT systems since the image reconstruction methods and image registration algorithms depend on the CBCT system.

## Conclusion

The imaging dose could be reduced to half or less with maintaining the registration accuracy in auto-bone and manual-soft tissue matchings with kV-CBCT.
